# A sustainable and expedited ‘One‐Stop’ prostate cancer diagnostic pathway to reduce environmental impact and enhance accessibility

**DOI:** 10.1002/bco2.447

**Published:** 2024-10-23

**Authors:** Lorenzo Storino Ramacciotti, Masatomo Kaneko, Severin Rodler, Muneeb Mohideen, Jie Cai, Gangning Liang, Manju Aron, Michelle Hopstone, Mariana C. Stern, Giovanni E. Cacciamani, Inderbir Gill, Andre Luis Abreu

**Affiliations:** ^1^ USC Institute of Urology, Catherine and Joseph Aresty Department of Urology, Keck School of Medicine University of Southern California Los Angeles California USA; ^2^ Center for Image‐Guided Surgery, Focal Therapy and Artificial Intelligence for Prostate Cancer, Keck School of Medicine University of Southern California Los Angeles California USA; ^3^ Department of Pathology, Keck School of Medicine University of Southern California Los Angeles California USA; ^4^ Department of Radiology, Keck School of Medicine University of Southern California Los Angeles California USA

**Keywords:** carbon footprint, One‐Stop pathway, prostate biopsy, prostate cancer

## Abstract

**Objective:**

To assess the carbon footprint, accessibility, and diagnostic performance of an expedited ‘One‐Stop’ prostate cancer (PCa) diagnostic pathway.

**Materials and methods:**

A total of 1083 consecutive patients undergoing magnetic resonance imaging (MRI) followed by transrectal ultrasound fusion‐guided prostate biopsy (PBx) were identified from a prospective database. The patients were divided according to the diagnostic pathway: One‐Stop, with MRI and same‐day PBx (3 hours apart), or Standard, with MRI followed by a second visit for PBx. Socioeconomic status was evaluated by the Distressed Communities Index (DCI) and the carbon footprint by the United States (U.S.) Environmental Protection Agency Greenhouse Gases Equivalencies Calculator.

**Results:**

Overall, 260 patients underwent the One‐Stop and 823 the Standard pathway. The One‐Stop patients lived farther from the hospital (163 vs. 23 km; *p* < 0.001), had lower socioeconomic status with DCI scores of 49 versus 30 (*p* < 0.001), and were more likely to be Latinos (21% vs. 13%, *p* < 0.001) compared to the Standard patients, respectively. The One‐Stop saved 69 575 km in round trips, over 16 tons of travel‐related CO_2_ emissions, and $8214 U.S. dollars. For patients with Prostate Imaging Reporting & Data System (PIRADS) 3–5, the clinically significant PCa detection (53% vs. 50%, *p* = 0.55) was similar for the One‐Stop and Standard pathways, respectively.

**Conclusions:**

The One‐Stop PCa diagnostic pathway reduces carbon footprint, distance travelled, and patient‐level cost while maintaining clinical outcomes comparable to the Standard pathway. It facilitates access to tertiary‐level care for minorities and underserved populations.

## 1 INTRODUCTION

The prostate cancer (PCa) diagnosis relies on high‐quality magnetic resonance imaging (MRI) examinations, followed by MRI‐informed prostate biopsies (PBxs). However, challenges emerge regarding accessibility and logistical considerations for individuals seeking this specialized care. This is further exacerbated for minorities and underserved populations, particularly those residing at considerable distances from tertiary healthcare centres. These factors can result in environmental impact due to extended travel distances, increased costs and limited access to services for distressed populations. Furthermore, disparities in healthcare access and financial toxicity can lead to delayed diagnoses and poorer outcomes.^1^, ^2^

Over 2 million PBx are performed annually in Europe and the United States (U.S.).^3^ This high volume of procedures poses a significant environmental impact.^4^ Traditional diagnostic pathways for PCa often involve multiple in‐person appointments, including initial consultations, MRI scans, and subsequent biopsies. Each step in this process not only consumes considerable time and resources but also contributes to the healthcare sector's carbon footprint.^4^

Healthcare is responsible for 4.4% of carbon dioxide (CO_2_) emissions worldwide.^5^ Therefore, it is crucial to reduce healthcare's greenhouse gas (GHG) emissions without compromising the quality of care. Various strategies for minimizing the carbon footprint of healthcare have been implemented, including reusable medical instruments, adopting practices to decrease waste production, and telemedicine.^6^, ^7^

We have previously described the implementation, safety, and feasibility of an expedited ‘One‐Stop’ pathway for PCa diagnosis in a small cohort.^8^, ^9^ Herein, we assess the carbon footprint, distance, cost savings, and accessibility, as well as the diagnostic performance of the One‐Stop PCa diagnostic pathway.

## 2 MATERIALS AND METHODS

### 2.1 Study population

Consecutive patients who underwent multiparametric MRI (mpMRI) followed by transrectal (TR) ultrasound fusion‐guided transperineal (TP) or TR PBx between January 2016 and March 2023 were identified from a prospectively maintained Institutional Review Board‐approved PBx database (No. HS‐13‐00663). Exclusion criteria were: (i) patients with mpMRI performed elsewhere; (ii) mpMRI acquired more than 6 months prior to the PBx; (iii) prior treatment for PCa; (iv) prior surgery for benign prostatic hyperplasia; (v) saturation PBx.

### 2.2 Diagnostic pathway

**FIGURE 1:**
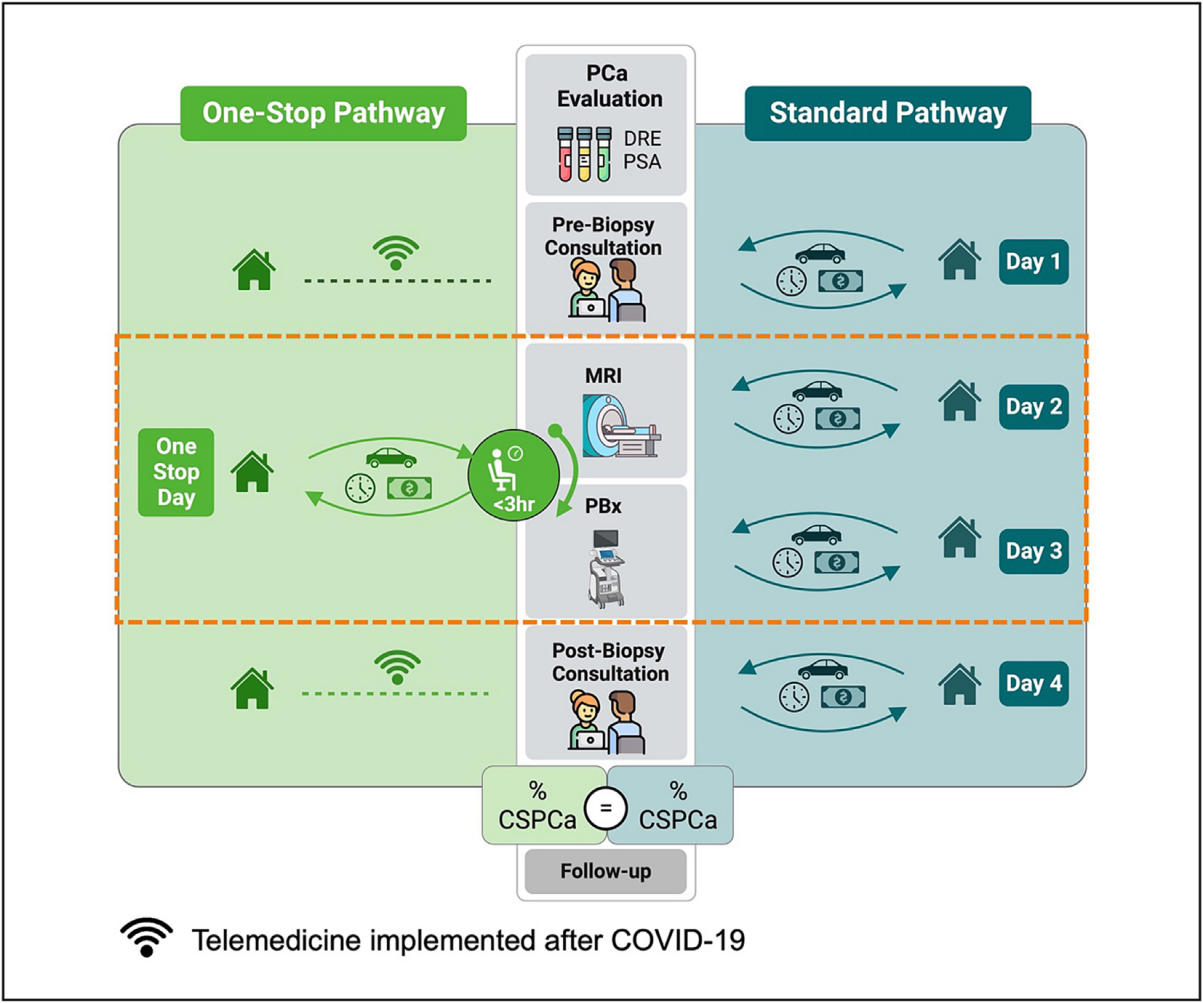
Comparative workflow of One‐Stop and Standard prostate cancer (PCa) diagnostic pathways. The One‐Stop pathway, established pre‐COVID‐19 pandemic, features a combined same‐day prostate magnetic resonance imaging (MRI) and biopsy (One‐Stop day). Post‐pandemic, this pathway incorporated telemedicine pre‐ and post‐biopsy consultations as part of its protocol. In contrast, the Standard pathway involves separate in‐person pre‐ and post‐biopsy consultations (Days 1 and 4), as well as separate appointments for prostate biopsy (PBx) and MRI (Days 2 and 3). The orange rectangle highlights the One‐Stop day and Days 2 and 3, which were the focus of the savings analysis in the present study. CSPCa, clinically significant prostate cancer; DRE, digital rectal examination.

Patients referred for PBx were offered two diagnostic pathway options on pre‐biopsy consultation: the expedited ‘One‐Stop’ pathway or the two‐visit ‘Standard’ pathway (Figure 1). The One‐Stop pathway consisted of acquiring a mpMRI followed by a PBx performed on the same day, therefore sparing one round trip from the patient's residence to the institution. The Standard pathway consisted of acquiring a mpMRI prior to the PBx but on separate days.

A standardized workflow protocol was developed and implemented for the One‐Stop pathway as follows: (i) prior notification was provided to the radiology team regarding One‐Stop patients and the MRI was ordered as stat reading; (ii) mpMRI were interpreted immediately following mpMRI acquisition; (iii) PBx was performed within 3 hours from the mpMRI acquisition to ensure sufficient time for adequate image processing and interpretation without imposing time constraints on radiologists. Both diagnostic procedures (MRI and PBx) were performed at the same institution. In response to the COVID‐19 pandemic restrictions, the One‐Stop pathway protocol incorporated telemedicine for pre‐ and post‐biopsy consultations.^8^ However, the present study did not consider these telemedicine consultations in its analysis, as it included patients from the pre‐pandemic period.

The One‐Stop pathway was specifically offered for patients who lived far from the facility or for those who expressed a preference for an expedited diagnostic pathway. The selection for the One‐Stop pathway was not influenced by any other demographics, clinical parameters, G or biopsy approach.^8^, ^9^

### 2.3 MRI acquisition and interpretation

All mpMRIs were acquired and interpreted at our institution in accordance with the Prostate Imaging Reporting & Data System (PIRADS) v2.0 or v2.1 by radiologists with over 5 years of expertise in prostate mpMRI reading.^10^, ^11^ The detailed MRI protocol has been previously described.^8^, ^9^, ^12^, ^13^, ^14^

### 2.4 Prostate biopsy protocol

All PBx were carried out transperineally or transrectally by a single urologist (ALA) using a three‐dimensional organ‐tracking elastic image fusion system (Trinity, Koelis®, Grenoble, France) and 18G needle biopsy as previously described.^9^, ^13^, ^14^, ^15^, ^16^ TP biopsies for both pathways (One‐Stop or Standard) were initiated in 2020 as the institution transitioned from TR to TP PBx.^13^, ^15^ All patients underwent 12–14 cores systematic biopsies (SBs), with a minimum of two additional targeted cores per each PIRADS 3–5 lesion. The PBx specimens were evaluated by a uropathologist according to the International Society of Urological Pathology (ISUP) guidelines.^17^ The pathology report followed its regular pacing as per the institution's standard. Clinically significant PCa (CSPCa) was defined as Grade Group ≥2.

### 2.5 Patients' socioeconomic status

The Distressed Communities Index (DCI) was used to assess patients' socioeconomic status (SES). It was developed by the Economic Innovation Group as a tool for measuring the economic well‐being of U.S. communities.^18^ It was derived from the Census Bureau's Business Patterns and American Community Survey 5‐Year Estimates for 2016–2020 and is a zip code‐based composite score of community education rates, poverty rate, unemployment, housing vacancies, median household income, change in employment, and change in business establishments. The DCI scores range from 0 (most prosperous) to 100 (most distressed), with higher scores indicating a lower SES. The One‐Stop and Standard pathways patients' 5‐digit zip codes were linked to the DCI database, and the DCI scores were assessed.

### 2.6 Race and ethnicity

Race and ethnicity were self‐reported according to U.S. National Institutes of Health standards as follows: Hispanic/Latino (Latino); non‐Hispanic Asian (Asian), non‐Hispanic Black or African American (Black), non‐Hispanic White (White), and Others (American Indian or Alaska Native; Native Hawaiian or Other Pacific Islander; and those who did not report or identify as any race or ethnicity).^19^, ^20^

### 2.7 Carbon footprint, greenhouse gases equivalencies, distance and cost calculation

The total travel distance was quantified and calculated in kilometres (km) according to the postal codes of the patient's residence and the clinic's location as previously described.^21^ This distance was then doubled to represent the complete round‐trip journey to and from the medical facility. All patients were assumed to travel round‐trip via automobile from their home address to the hospital per each visit. CO_2_ emissions saved for vehicle travel and equivalencies were calculated using the U.S. Environmental Protection Agency (EPA) emissions calculator, which estimates 390 g of CO_2_ emissions per vehicle per mile travelled.^22^ Travel costs were estimated by multiplying the round‐trip distance by the mean of the 2016–2023 reimbursable Federal Standard Mileage rates for medical and moving purposes of 19 cents per mile, which applies to electric, hybrid‐electric, gasoline, and diesel‐powered vehicles.^23^, ^24^ All costs were reported as U.S. dollars. Patients who did not live in California were excluded from these analyses as the variability of airplane travel was not considered. This ensured accurate measurement of travel distance, CO_2_ emissions, and costs based solely on ground transportation.

### 2.8 Endpoints

The primary endpoints were the savings from travel‐related CO_2_ emissions, travel distance, and costs of the One‐Stop pathway during the study period.

The secondary endpoints were the detection of CSPCa for One‐Stop versus Standard pathways and the complication rates. SES and race/ethnicity were also compared between groups. Complications were recorded and reported up to 30 days post‐biopsy according to the Clavien–Dindo (CD) classification system.^25^ Intra‐ and postoperative complications were recorded and reported.^26^

### 2.9 Statistical analysis

The Wilcoxon rank sum test was used for continuous variables, and Pearson's chi‐square or Fisher exact test was used for categorical variables. Univariable and multivariable logistic regression analyses were performed to correlate clinical parameters related to CSPCa detection. Statistical analyses were conducted using SAS version 9.4 (SAS Institute Inc., Cary, NC, USA). A two‐sided *p*‐value of <0.05 was considered statistically significant.

## 3 RESULTS

**FIGURE 2:**
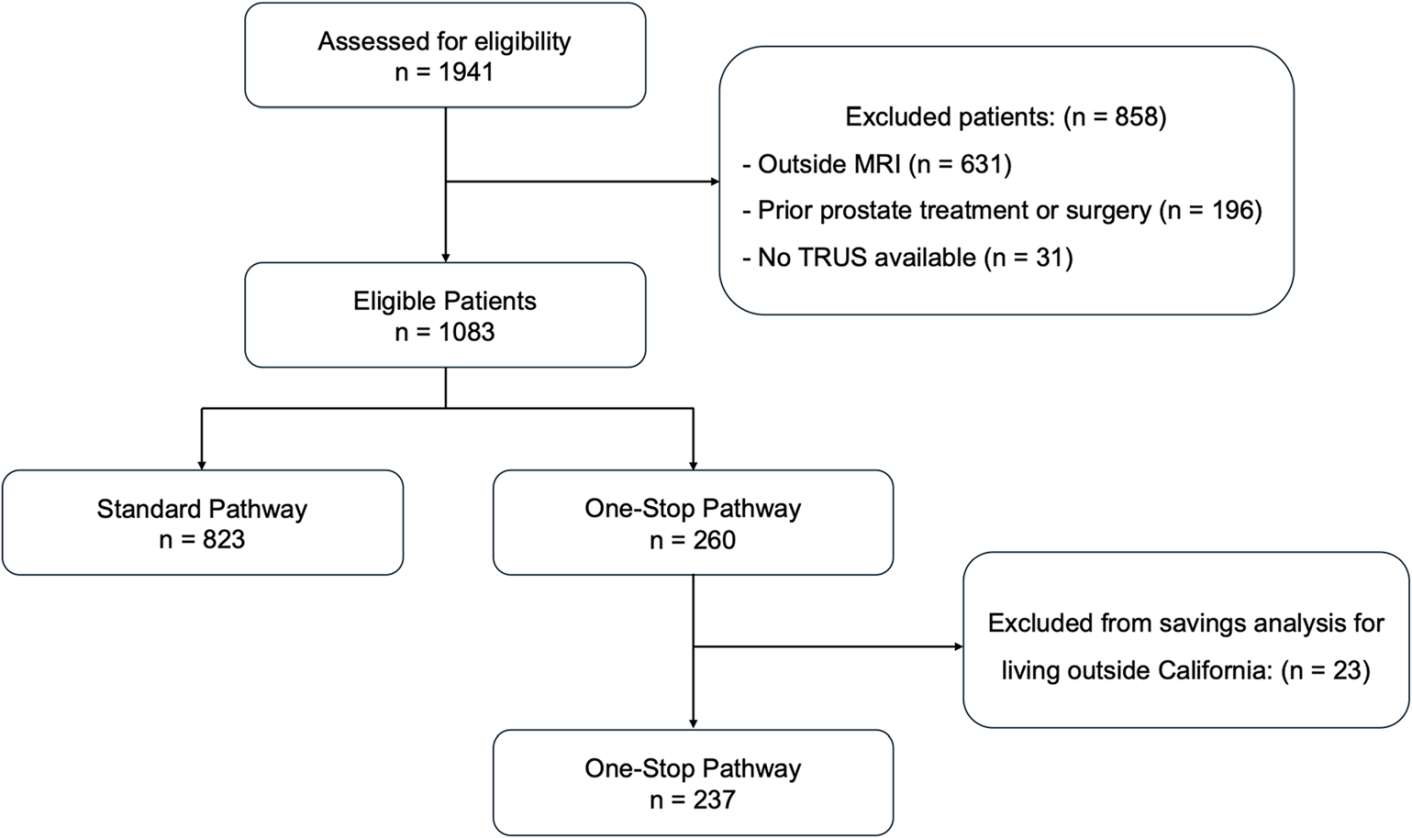
Diagram reporting the eligible patients and reasons for exclusion.

**FIGURE 3:**
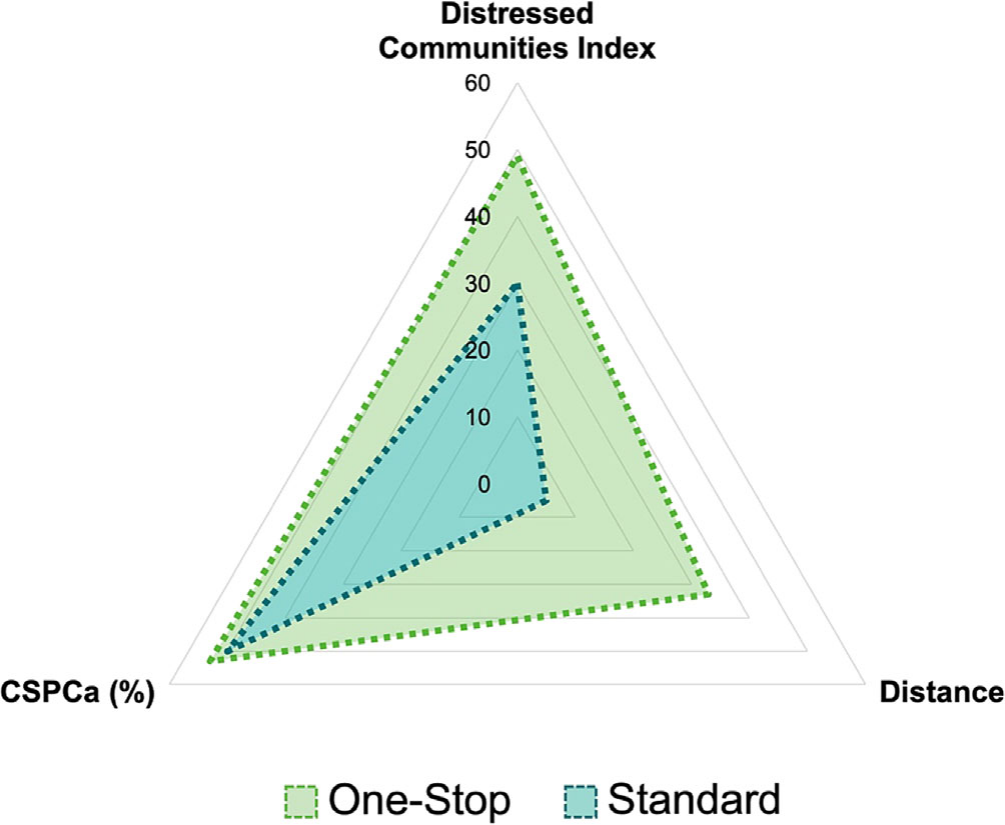
Radar chart representing the comparison between the One‐Stop and Standard pathways for clinically signficant prostate cancer (CSPCa) diagnosis, distance travelled, and soscioeconomoci status. The One‐Stop cohort travelled a sevenfold greater median round‐trip distance from their residence to the hospital (326 vs. 46 km; *p* < 0.001) and exhibited a lower socioeconomic status, as indicated by a higher median Distressed Community Index score of 49 vs. 30 (*p* < 0.001). Among patients with Prostate Imaging Reporting & Data System (PIRADS) 3–5, the detection rates of clinically significant prostate cancer (CSPCa) were comparable between the One‐Stop and Standard cohorts, at 53% and 50%, respectively (*p* = 0.55). Distance: the median round‐trip distance (km, 10^−1^) from the patient's residence to the hospital.

A total of 1083 patients met the inclusion/exclusion criteria, with 260 patients in the One‐Stop and 823 in the Standard pathway groups (Figure 2). The median age (65 vs. 66 years; *p =* 0.2), PSA (6.5 vs. 6.6 ng/mL; *p* = 0.48), PSA density (0.11 vs. 0.13 ng/mL^2^; *p* = 0.07), prostate volume (54 vs. 54 cc; *p =* 0.7) and PIRADS 3–5 distribution (67% vs. 73%; *p* = 0.07) were similar between One‐Stop and Standard pathway groups, respectively (Table 1). The One‐Stop patients lived farther from the hospital (median round‐trip distance, 326 vs. 46 km; *p* < 0.001), had lower SES (median DCI scores, 49 vs. 30; *p* < 0.001), and were more likely to be Latinos (21% vs. 13%; *p* < 0.001) compared to the Standard patients, respectively (Figure 3). The median time from MRI to PBx was shorter for One‐Stop patients (0 vs. 12 days; *p* < 0.001).

**TABLE 1:** Baseline characteristics of MRI/TRUS fusion prostate biopsy.

	One‐Stop pathway	Standard pathway	*p*
No. of patients, *n* (%)	260	823	
Age, year, median (IQR)	65 (60–70)	66 (61–71)	0.2
Carlson comorbidity index, median (IQR)	1 (0–2)	1 (0–2)	0.9
Family history PCa, *n* (%)	65 (28)	213 (28)	0.8
Race and ethnicity, *n* (%)			<0.001
Asian	12 (4.6)	95 (12)	
Black or African American	8 (3.1)	52 (6.3)	
Latino or Hispanic	54 (21)	103 (13)	
Other or not reported	46 (18)	106 (13)	
White	140 (54)	467 (57)	
Distress Communities Index quintiles, *n* (%)			<0.001
Prosperous	63 (25.8)	267 (34)	
Comfortable	40 (16.4)	206 (26.2)	
Mid‐tier	42 (17.2)	159 (20.3)	
At RiskQuintile	55 (22.5)	100 (12.7)	
Distressed	44 (18)	53 (6.8)	
Distress Communities Index Score (0–100), median (IQR)	49 (20–74)	30 (14–54)	<0.001
Biopsy history, *n* (%)			0.08
Naïve	136 (53)	480 (59)	
Negative	58 (23)	186 (23)	
In active surveillance	64 (25)	152 (19)	
PSA, ng/mL, median (IQR)	6.5 (4.8–9.2)	6.6 (4.8–9.9)	0.5
PSA density, ng/mL^2^, median (IQR)	0.11 (0.07–0.18)	0.13 (0.08–0.20)	0.07
Suspicion for PCa on DRE, *n* (%)	65 (25)	202 (25)	0.9
Time from MRI to biopsy, days, median (IQR)	0	12 (6–27)	<0.001
Home‐hospital round trip distance, km, median (IQR)	326 (80–474)	46 (28–100)	<0.001
Prostate volume, cc, median (IQR)	54 (37–77)	54 (38–76)	0.7
No. MRI lesions, median (IQR)	1 (0–2)	1 (0–2)	0.5
MRI index lesion location^a^, *n* (%)			
Anterior	57 (22)	182 (22)	1.0
Posterior	135 (52)	474 (58)	0.1
Base	59 (23)	176 (21)	0.7
Mid	114 (44)	380 (46)	0.5
Apex	60 (23)	209 (25)	0.5
MRI index lesion size^a^, mm, median (IQR)	13 (9–19)	12 (9–17)	0.2
PIRADS score, *n* (%)			0.07
PIRADS 1–2	85 (33)	221 (27)	
PIRADS 3	77 (30)	281 (34)	
PIRADS 4	51 (20)	200 (24)	
PIRADS 5	47 (18)	121 (15)	

Abbreviations: CSPCa, clinically significant PCa (grade group ≥2); DRE, digital rectal examination; IQR, interquartile range; MRI, magnetic resonance imaging; No., number; PCa, prostate cancer; PIRADS, Prostate Imaging Reporting & Data System; TRUS, transrectal ultrasound.

^a^ Index lesion (highest PIRADS, then the largest lesion).

### 3.1 One‐Stop pathway savings

**FIGURE 4:**
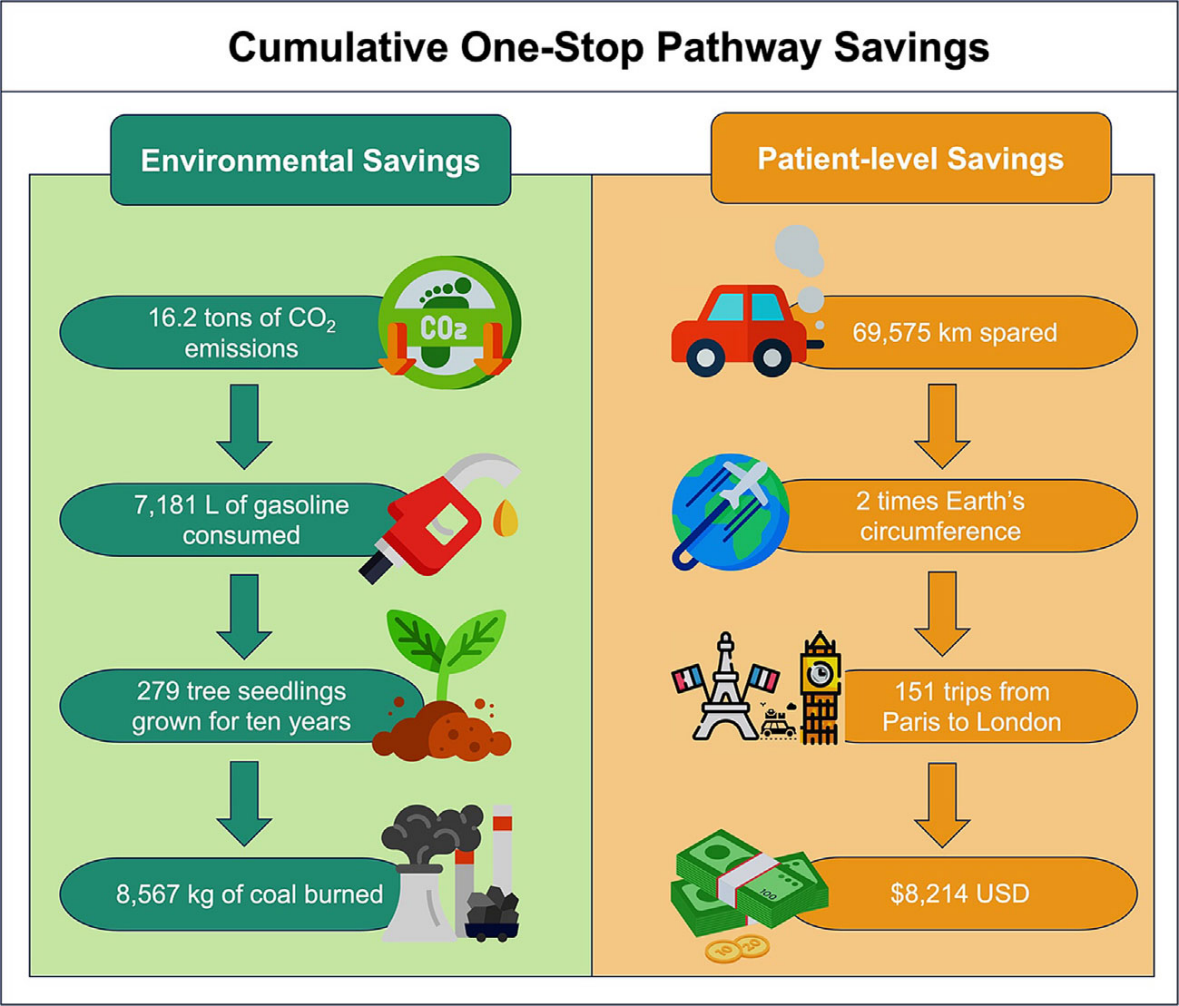
Cumulative One‐Stop pathway environmental and patient‐level savings and their respective equivalencies.

Twenty‐three patients from the One‐Stop cohort were excluded from the savings analysis as they did not live in California (Figure 2). The One‐Stop pathway resulted in an estimated total savings of 69 575 km in round trips, equivalent to circling the Earth nearly two times (Figure 4). This also led to an estimated reduction of 16 861 kg in travel‐related CO_2_ emissions, which is comparable to the emissions from consuming 7181 L of gasoline, burning 8567 kg of coal, or the carbon sequestration achieved by growing 279 tree seedlings for 10 years. Overall, each One‐Stop patient avoided a median (interquartile range [IQR]) of 310 (69–472) km in round trips, saving 75 kg (17–114) of CO_2_ emissions. This is equivalent to not consuming 31.8 L of gasoline, not burning 38.1 kg of coal, or the carbon sequestration of 1.8 tree seedlings grown for 10 years. Regarding travel‐related costs, the One‐Stop cohort saved a total of $8214, with a median of $36.6 (8.2–55.7) per patient.

### 3.2 Clinical outcomes

**FIGURE 5:**
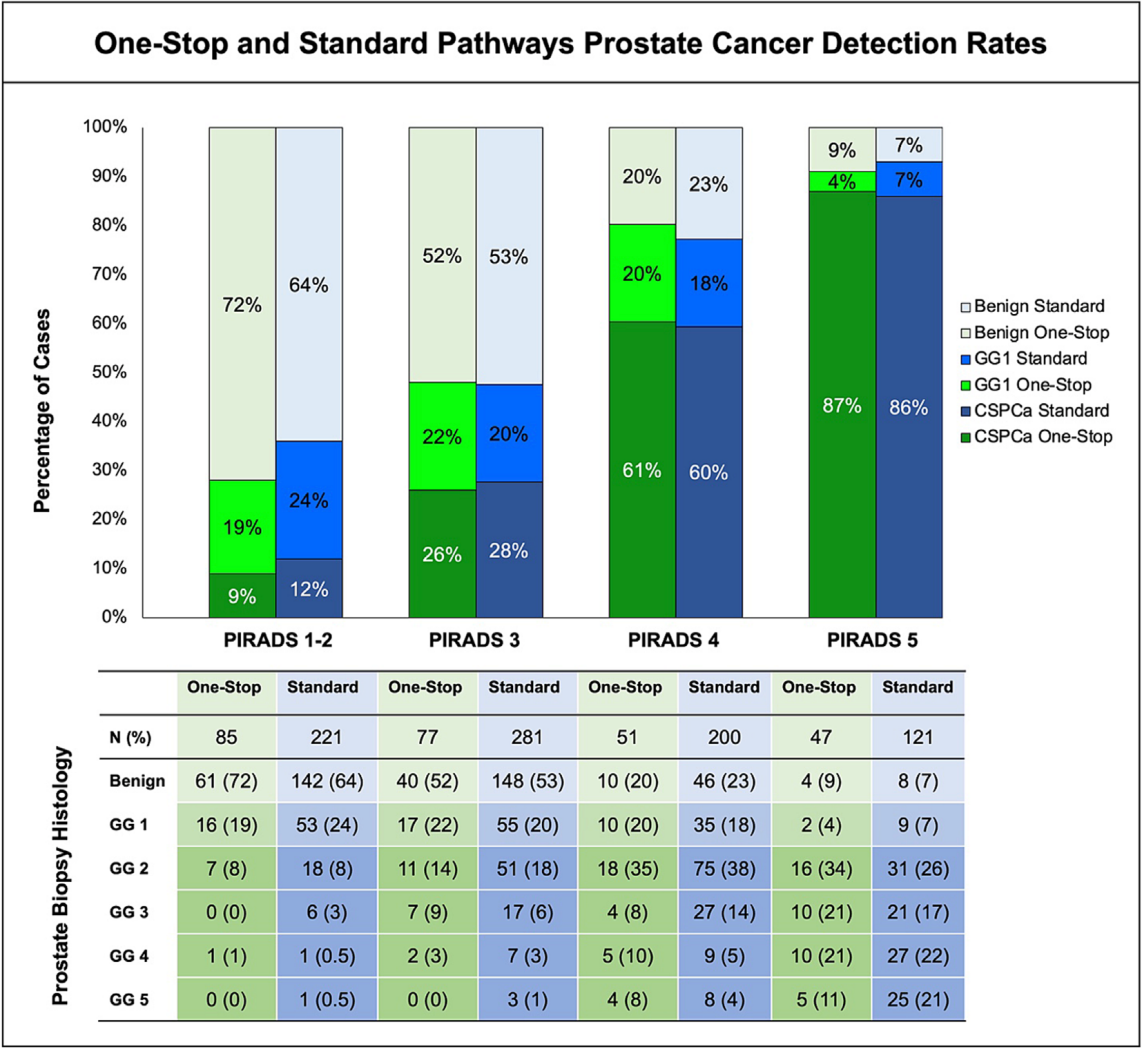
Prostate cancer (PCa) and clinically significant PCa (CSPCa) detection rates for the One‐Stop and Standard pathways according to the Prostate Imaging Reporting & Data System (PIRADS). GG, grade group.

For patients with PIRADS 3–5 lesions identified on mpMRI, the PCa (69% vs. 67%; *p* = 0.5) and CSPCa (53% vs. 50%; *p* = 0.55) detection rates were similar for One‐Stop and Standard groups, respectively (Figure 5). On a multivariable logistic regression, age, negative PBx history, PSA, digital rectal examination findings, prostate volume, PIRADS 3–5, and the number of targeted cores taken were independent predictors for CSPCa detection, but the diagnostic pathway and biopsy approach were not (Table S1). The median number of targeted biopsy cores sampled (4 vs. 4; *p* = 0.4) and positive targeted samples (2 vs. 2; *p* = 0.5) were similar between the One‐Stop and Standard pathways, respectively. The sensitivity, negative predictive value, and accuracy of MRI for detecting CSPCa on PBx were similar for both pathways (Table S2).

Perioperative complications were low and not statistically significantly different between One‐Stop and Standard pathways (2.7% vs. 2.2% *p* = 0.6), respectively (Table S3). Two patients experienced intraoperative complications in the Standard pathway: a vasovagal reflex and a case of rectal bleeding during TR anaesthesia. Both intraoperative complications required no intervention, with both patients being discharged after the procedure. No intraoperative complications occurred in the One‐Stop pathway group.

## 4 DISCUSSION

In this prospective database study, the One‐Stop expedited PCa diagnostic pathway in a diverse patient population substantially saved carbon emissions, distance travelled, and patient‐level cost. The One‐Stop cohort included a higher number of patients from underserved communities who lived farther away from the hospital. Yet, the CSPCa detection rates were similar to the Standard pathway. The One‐Stop protocol promotes inclusion, accessibility, equity, and sustainability without compromising quality of care or clinical outcomes.

A recent study evaluating the PCa detection pathway emphasized the different strategies and the importance of reducing CO_2_ emissions by optimizing the PCa diagnosis workflow.^4^ However, this study relied on hypothetical scenarios, lacking tangible evidence of its clear applicability based on real‐world data. Herein, to the best of our knowledge, we report the first study in urology that utilizes metrics and actual data to evaluate the carbon footprint of a PCa diagnostic workflow. Previous researchers mainly focused on product consumption and, therefore, life‐cycle assessment to identify strategies to lower the carbon footprint. For example, using disposable devices for cystoscopy has been demonstrated to reduce carbon footprint.^27^ Differently, we focused on optimizing the logistics and workflow.

Researchers from the United Kingdom evaluated a Rapid Assessment for Prostate Imaging and Diagnosis (RAPID) diagnostic pathway^28^ designed to reduce healthcare burdens and standardize pre‐biopsy MRI. Like our study, RAPID focused on facilitating prompt access to high‐quality MRI and the potential for immediate same‐day PBx. A primary goal of RAPID was the widespread implementation of PCa screening MRIs. In our study, all patients received pre‐biopsy MRI in both diagnostic pathways. Prebiopsy MRI has been widely demonstrated to significantly reduce the costs associated with PCa diagnostics.^29^, ^30^ Furthermore, the One‐Stop pathway's aim extends beyond expediting the process. It aims to simplify and enhance accessibility while simultaneously speeding up the diagnostic pathway. Nevertheless, the time from MRI to PBx was shorter for One‐Stop patients (0 vs. 12 days, *p* < 0.001). The RAPID researchers also assessed patient satisfaction, with 96% rating the experience as ‘very good or good’. Our study did not assess patient satisfaction. Future prospective studies should include patient satisfaction surveys to compare expedited and non‐expedited PCa diagnostic pathways.

The primary contributor to greenhouse gas (GHG) emissions in in‐person appointments is the distance travelled.^31^ In the current study, the method for assessing CO_2_ emission reduction centred around travel reduction, similar to studies that evaluate the carbon footprint of telemedical consultations.^6^ However, unlike studies that compare the carbon footprint reduction of telemedicine to in‐person visits, where the intervention itself is modified, the One‐Stop pathway does not alter the standard of care. In this cohort, patients saved a total of 69 575 km of round trips, equivalent to driving from Paris to London 151 times.

The SES of patients was assessed using the DCI, a tool previously utilized in surgical literature for predicting outcomes and aiding in risk stratification.^32^ Patients in the One‐Stop cohort resided sevenfold farther from the institution and had significantly higher DCI scores than patients from the Standard pathway, indicating that One‐Stop patients have lower SES and reside in more distressed communities. Furthermore, patients with PCa who have lower SES, limited educational attainment, and those residing in rural areas have been linked to increased financial burdens that adversely impact PCa survival.^2^, ^33^ The One‐Stop pathway provided patients from underserved communities access to attaining care in a tertiary centre while reducing the cost, distance travelled, and CO_2_ emissions. This is extremely relevant in light of the ongoing trend of rural hospital closures in the U.S., which has led to an increasing number of individuals living more than a 60‐min drive from a primary hospital.^34^

Calculating the per‐patient cost is intricate, given the myriad variables involved. This complexity arises not only from direct expenses such as parking, food, and fuel but also due to patients often not attending their in‐person appointments. This not only increases the incidental costs but also amplifies the indirect financial impact. Furthermore, these indirect costs can influence patient behaviour, possibly leading to missed appointments or delayed care, especially in lower‐income groups. Therefore, the estimated savings identified in this study might indeed be conservative.

The diagnosis of PCa is continuously evolving. More recently, we fully transitioned from TR to TP PBx approach. Nevertheless, we could maintain the One‐Stop workflow because the TP PBx are routinely performed in the outpatient clinic under local anaesthesia, using similar setup, equipment, and personnel as the TR approach.^13^, ^15^ Performing TP PBx in the clinic is not only essential due to the limited availability of operating rooms (ORs) but also beneficial, as OR‐based procedures contribute significantly to GHG emissions, are costlier, and require a larger number of specialized healthcare providers.^7^ Furthermore, the TP approach contributes to antibiotic stewardship and potentially reduces infectious complications.^35^ Regardless of the biopsy approach, complications in this study were low and not statistically significantly different between the One‐Stop and Standard pathways, with similar CSPCa detection rates observed between the two pathways.

This study has limitations. First, all patients were assumed to have completed round trips by automobile transport without considering the possibility of airfare. Patients were not surveyed regarding their type of transportation; therefore, the U.S. EPA calculator for an average gasoline‐powered passenger car was used as previously described.^6^ These potential confounding factors would apply to both groups and, therefore, the comparison would still be valid. In California, there is a clear gap and inequity in the adoption of electric vehicles, with lower SES and Black and Latino patients having less access to electric vehicles.^36^, ^37^, ^38^ Our study had almost twice as many Latinos in the One‐Stop pathway (21% vs. 13%); thereby, if there is to be expected a difference in the proportion of electric vehicles used, the Standard pathway patients would have been favoured. Another limitation is that the current model does not take into consideration other potential contributors to CO_2_ emissions other than travel distance. Cost savings were possibly underestimated because only the deductible mileage rate was considered without considering extra costs such as those related to complication management post‐biopsy (i.e., re‐admissions). Nonetheless, these metrics provide objective measures and avoid reliance on hypothetical scenarios. All biopsies in both pathways were conducted at a single tertiary centre, thus limiting the generalizability of the results.

## 5 CONCLUSIONS

The One‐Stop PCa diagnostic pathway reduces carbon footprint, distance travelled, and patient‐level cost while maintaining clinical outcomes comparable to the Standard pathway. Furthermore, it enables minorities and underserved populations to access tertiary‐level care, especially those residing at greater distances from medical centres. The One‐Stop PCa pathway is a sustainable, efficient, and accurate approach for PCa diagnostics.

## AUTHOR CONTRIBUTIONS

*Concept*: Lorenzo Storino Ramacciotti, Masatomo Kaneko and Andre Luis Abreu. *Data acquisition*: Lorenzo Storino Ramacciotti, Masatomo Kaneko, Severin Rodler and Muneeb Mohideen. *Statistical analysis*: Jie Cai. *Supervision*: Gangning Liang, Manju Aron, Mariana C. Stern, Michelle Hopstone, Giovanni E. Cacciamani, Inderbir Gill and Andre Luis Abreu. *Writing (original draft)*: Lorenzo Storino Ramacciotti, Severin Rodler and Andre Luis Abreu. *Writing (review and editing)*: Lorenzo Storino Ramacciotti, Masatomo Kaneko, Severin Rodler, Muneeb Mohideen, Gangning Liang, Manju Aron, Mariana C. Stern, Michelle Hopstone, Giovanni E. Cacciamani, Inderbir Gill and Andre Luis Abreu.

## CONFLICT OF INTEREST STATEMENT

S.R. receives consultancy fees from Merck, MSD and Novartis and has equity in Rocketlane Medical Ventures GmbH. I.G. has equity interest in OneLine Health and Karkinos. A.L.A. is a consultant for Koelis, a speaker for EDAP and a proctor for Sonablate. Other authors do not have any competing interests.

## Supporting information


**Table S1.** Univariable and Multivariable analyses for clinically significant prostate cancer detection on MRI/TRUS fusion prostate biopsy.
**Table S2.** Diagnostic accuracy of the magnetic resonance imaging for clinically significant prostate cancer detection on prostate biopsy.
**Table S3.** Perioperative complications for One‐Stop vs Standard MRI/TRUS fusion prostate biopsy.
