# Supplementary material for: A sustainable and expedited ‘One‐Stop’ prostate cancer diagnostic pathway to reduce environmental impact and enhance accessibility
Source: BJUI Compass. 2024 Oct 23;5(12):1278–87. doi: 10.1002/bco2.447 (PMC11685178; doi:10.1002/bco2.447)
Supplement: Supplementary file 1 — Table S1. Univariable and Multivariable analyses for clinically significant prostate cancer detection on MRI/TRUS fusion prostate biopsy. Table S2. Diagnostic accuracy of the magnetic resonance imaging for clinically significant prostate cancer detection on prostate biopsy. Table S3. Perioperative complications for One‐Stop vs Standard MRI/TRUS fusion prostate biopsy. [file BCO2-5-1278-s001.docx]

**Supplementary material**

**Supplementary Table 1 –** Univariable and Multivariable analyses for clinically significant prostate cancer detection on MRI/TRUS fusion prostate biopsy.

|  | **Univariable** | | **Multivariable** | |
| --- | --- | --- | --- | --- |
| **Variables** | **OR** | **CI (95%)** | **OR** | **CI (95%)** |
| **Age, year** | 1.06 | 1.05-1.08 | 1.07 | 1.04-1.09 |
| **Family History PCa** | 0.91 | 0.68-1.21 |  |  |
| **Biopsy history** |  |  |  |  |
| **Previous Negative biopsy vs Naïve** | 0.45 | 0.32-0.62 | 0.57 | 0.38-0.85 |
| **Previous Positive biopsy vs Naïve** | 1.17 | 0.85-1.6 | 1.23 | 0.84-1.82 |
| **PSA, ng/ml** | 1.05 | 1.03-1.08 | 1.08 | 1.05-1.11 |
| **PSA density*, ng/ml^2^** | 1.01 | 1.01-1.02 |  |  |
| **Race and Ethnicity** |  |  |  |  |
| **Asian vs NH-White** | 0.98 | 0.64-1.48 |  |  |
| **Latino vs NH-White** | 0.86 | 0.6-1.23 |  |  |
| **Black vs NH-White** | 1.61 | 0.95-2.74 |  |  |
| **Others vs NH-White** | 0.86 | 0.59-1.23 |  |  |
| **DRE, suspicious vs non-suspicious** | 4.55 | 3.4-6.14 | 3.35 | 2.34-4.82 |
| **Prostate Volume, cc** | 0.98 | 0.98-0.99 | 0.98 | 0.97-0.98 |
| **No. MRI lesions** | 1.77 | 1.54-2.04 |  |  |
| **MRI lesion size, mm** | 1.07 | 1.04-1.09 |  |  |
| **PIRADS 3-5 vs PIRADS 1-2** | 8.19 | 5.66-12.2 | 3.46 | 2.01-6.05 |
| **No. TB cores taken** | 1.36 | 1.29-1.45 | 1.18 | 1.08-1.29 |
| **Prostate biopsy approach TP vs TR** | 1.45 | 1.1-1.9 | 1.40 | 0.98-2.00 |
| **One-Stop vs Standard pathway** | 0.95 | 0.71-1.26 | 0.91 | 0.64-1.28 |
| PIRADS, Prostate Imaging Reporting and Data System; MRI, magnetic resonance imaging; OR, odds ratio; CI, confidence interval; PCa, prostate cancer; CSPCa, Clinically significant PCa (Grade Group > 1); DRE, digital rectal examination; DRE, digital rectal examination; NH, non-Hispanic.  *PSA density was calculated per 0.01 unit. | | | | |

**Supplementary Table 2 –** Diagnostic accuracy of the magnetic resonance imaging for clinically significant prostate cancer detection on prostate biopsy.

|  | One-Stop | Standard | *P* |
| --- | --- | --- | --- |
| Sensitivity (CI 95%) | 0.92 (0.84-0.96) | 0.92 (0.88-0.94) | 0.44 |
| Specificity (CI 95%) | 0.48 (0.4-0.56) | 0.39 (0.35-0.44) |  |
| Negative Predictive Value (CI 95%) | 0.91 (0.82-0.96) | 0.88 (0.83-0.92) |  |
| Positive Predictive Value (CI 95%) | 0.52 (0.45-0.6) | 0.5 (0.46-0.54) |  |
| Accuracy | 0.65 | 0.6 |  |

**Supplementary Table 3. Perioperative complications for One-Stop vs Standard MRI/TRUS fusion prostate biopsy**

|  | **One-Stop** | **Standard** | ***P*** |
| --- | --- | --- | --- |
| **Number of patients** | 260 | 823 |  |
| **Complications, number of patients, n (%)** |  |  |  |
| **Any complication** | 7(2.7)** | 18 (2.2)^+^ | 0.6 |
| **Infectious*** | 7 (2.7) | 8 (1.0) |  |
| **Urinary retention** | 1 (0.4) | 7 (0.9) |  |
| **Transient ischemic attack** | - | 1 (0.1) |  |
| **Vasovagal reflex** | - | 1 (0.1) |  |
| **Prolonged hematuria** | - | 2 (0.2) |  |
| **Rectal bleeding** | - | 2 (0.2) |  |
| **Clavien-Dindo Grade^++^, n (%)** |  |  | 0.12 |
| **I** | - | 5 (0.6) |  |
| **II** | 6 (2.3) | 7 (0.9) |  |
| **III** | 1 (0.4) | 1 (0.1) |  |
| **IV** | - | 3 (0.4) |  |
| *Infectious complications include urinary tract infections and urosepsis **One patient experienced urinary retention (IIIb) and urosepsis 17 days after the procedure (II)  + One patient experienced urinary retention (II), urinary tract infection (II), and urosepsis (IV)  ++ Two complications were not graded with the Clavien-Dindo classification as they occurred intraoperatively. One was a vasovagal reflex, and the other was a case of rectal bleeding during transrectal anesthesia. | | | |
